# Understanding turbulent free-surface vortex flows using a Taylor-Couette flow analogy

**DOI:** 10.1038/s41598-017-16950-w

**Published:** 2018-01-16

**Authors:** Sean Mulligan, Giovanni De Cesare, John Casserly, Richard Sherlock

**Affiliations:** 1 Department of Civil Engineering and Construction, Institute of Technology Sligo, Sligo, Ireland; 2 Laboratory of Hydraulic Constructions (LCH), Ecole Polytechnique Fédérale de Lausanne (EPFL), CH-1015 Lausanne, Switzerland; 3 Department of Civil Engineering and Construction, Institute of Technology Sligo, Sligo, Ireland; 4 Centre for Precision Engineering, Materials and Manufacturing Research and School of Science, Institute of Technology Sligo, Sligo, Ireland

## Abstract

Free-surface vortices have long been studied to develop an understanding of similar rotating flow phenomena observed in nature and technology. However, a complete description of its turbulent three-dimensional flow field still remains elusive. In contrast, the related Taylor-Couette flow system has been well explicated which classically exhibits successive instability phases manifested in so-called Taylor vortices. In this study, observations made on the turbulent free-surface vortex revealed distinguishable, time-dependent “Taylor-like” vortices in the secondary flow field similar to the Taylor-Couette flow system. The observations were enabled by an original application of 2D ultrasonic Doppler velocity profiling complemented with laser induced fluorescence dye observations. Additional confirmation was provided by three-dimensional numerical simulations. Using Rayleigh’s stability criterion, we analytically show that a wall bounded free-surface vortex can indeed become unstable due to a centrifugal driving force in a similar manner to the Taylor-Couette flow. Consequently, it is proposed that the free-surface vortex can be treated analogously to the Taylor-Couette flow permitting advanced conclusions to be drawn on its flow structure and the various states of free-surface vortex flow stability.

## Introduction

Concentrated vortices are fascinating yet highly complicated flow phenomena which have deservedly been studied for centuries in the contexts of nature and technology^1–4^. The classic, free-surface draining vortex, or the so-called “bath-tub” vortex is among the most captivating of the families of vortex flows owing to the highly familiar yet unique depression of the free-surface accompanied by rotating surface ripples and a distinct appearance of “swirl”. However, beneath the surface and hidden to the eye of the observer is an intricate assembly of space and time dependent flow processes which still lack complete scientific elucidation^5^.

In general, free-surface vortices (FSVs) result from natural or artificial field circulation *Γ*_∞_ conditions generating a strong primary tangential velocity field *v*_*θ*_(*r*) characterised by turbulence through the vortex Reynolds number Re_Γ_ = *Γ*_*r*_/*ν* and rotational strength by the circulation number N_Γ_ = *dΓ*_∞_/*Q*. They can exist as either a ‘strong’ full air core or a ‘weak’ collapsed air core type which transition as a free-surface instability at the critical submergence *S*_*c*_^5–8^. The primary tangential velocity field *v*_*θ*_(*r*)in the free-surface vortex is superimposed on a more complex secondary flow field comprising the radial *v*_*r*_ and axial *v*_*z*_ velocity field which are responsible for ensuring continuity of flow *Q* through the system^9^. In two seminal studies undertaken independently on turbulent vortices by Anwar^10^ and Daggett and Keulegan^9^ it was discovered that the bulk of radial and axial flows were strongly localised to thin layers close to the base of the vortex, the free-surface and the air core region respectively and both studies suggested that it was the flow *Q* “*that provides energy to maintain an open vortex*”^9^. This was pivotal in providing an understanding of how full air core vortices maintain stability. Andersen *et al*.^5,6^, in their contribution on the “*anatomy of the bath*-*tub vortex*” also scrutinised the secondary flow field and attempted to decipher its composition, albeit for the weak laminar vortex flow. In their study^5,6^ they observed similar localised well-structured flow layers at low Reynolds number Re_Γ_ = 3 × 10^3^. At higher Reynolds number flows (Re_Γ_ = 7 × 10^4^), Echávez & McCann^11^ also noted similar flow layers at the boundaries and observed the appearance of well defined “hood-like” structures in the secondary flow field sub_-_surface depths. When dealing with turbulent vortices at Re_Γ_ = 8 × 10^5^, Anwar^10^ commented on the observation of aluminium particles suspended in the flow being drafted upwards near the core and thrown radially outwards indicating more intricate spatio-temporal flow behaviour at play in this sub-surface region. He also stated^12^ that the tangential velocity distribution *v*_*θ*_(*r*)differs from the laminar case due to the onset of turbulence triggered by the localised instability of curved flow as originally projected by Scorer^13^ who also pointed out that turbulence is attributed to large variations in axial flow in this region. It is clear from this that the turbulent FSV introduces higher degrees of complexity to the secondary flow field that demands a more complete analytical and experiential understanding.

**Figure 1:**
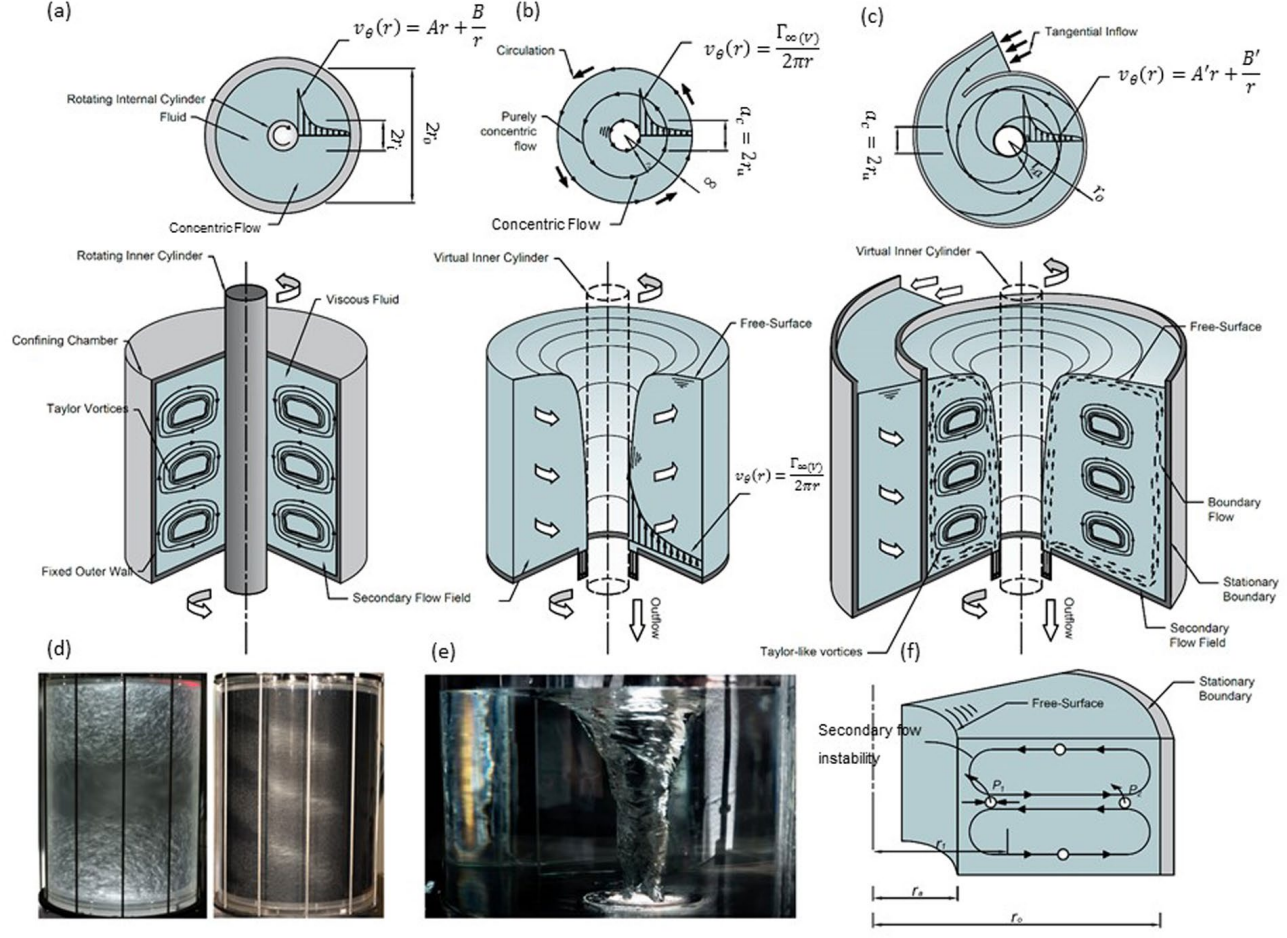
The analogue between secondary flow in the (**a**) Taylor-Couette flow (TCF) system (**b**) a laminar free-surface vortex (FSV) and (**c**) a turbulent vortex flow in a vortex chamber. (**d**) and (**e**) are images of the TCF and the FSV. In (**a**) the classic Taylor-Couette flow system is outlined where the internal cylinder of diameter 2 *r*_*i*_ is rotating at Ω_*i*_ and the external chamber is stationary. Rotation of the inner cylinder introduces centrifugal instabilities in the secondary flow field, which manifest as Taylor-vortices. On the other hand, Figure (**b**) and (**c**) outlines the strong full air core laminar and turbulent free-surface vortex structure, which receives energy by inflow to impart rotation or circulation *Γ*_∞_ on the flow field resulting in a depression of the free-surface around the outlet producing an air core of diameter *a*_*c*_ = 2*r*_*a*_. Taylor-like vortices superimposed on the flow processes outlined by Anwar^10^ and Daggett and Keulegan^9^ are presented in the secondary flow field of the vortex chamber together with an upwards flow in the far field as observed in this study. Figure 1 (**f**) provides a schematic example of ‘particle swaps’ of particles *P*_1_ and *P*_2_ demonstrating how the flow can become unstable as a result of the centrifugal driving force. The analogy between the Taylor-Couette and the free-surface vortex is realised if one replaces the air core *a*_*c*_ region of the free-surface vortex with a virtual inner cylinder 2 *r*_*i*_ rotating at the speed of the air core. In this way, equations representing the free-surface vortex flow field can be replaced with the angular velocity conditions of the virtual cylinder to yield equations for the TCF system. TCF flow image courtesy of Michael J. Burin^63^ (M.J. Burin, CSU San Marcos (2010)).

A relative of the FSV in the family of rotating flows is the classic Taylor–Couette flow (TCF) of an incompressible, viscous fluid in the gap between two concentric rotating cylinders of radii *r*_*i*_, *r*_*o*_ (*r*_*o*_ > *r*_*i*_) and height *l*^14–16^ as described in Fig. 1a. The control parameters of the system are generally taken^17^ as the radius ratio *η* = *r*_*i*_/*r*_*o*_, the cylinder angular velocity ratio *μ* = Ω_*o*_/Ω_*i*_, the aspect ratio *ζ* = *l*/(*r*_*o*_ − *r*_*i*_) and the Reynolds number Re defined by the Taylor number by Ta $=\,{r}_{i}{{{\rm{\Omega }}}_{i}}^{2}{({r}_{o}-{r}_{i})}^{3}/{\nu }^{2}$ which drives the flow by shear from the cylinder with an angular velocity of of Ω_*i*_^18^. The tangential velocity between the cylinders then takes the form of:

1 $${v}_{\theta }=V(r)=Ar+\frac{B}{r}$$

where the no-slip (shear driven) boundary conditions are:

2 $$A={{\rm{\Omega }}}_{i}\frac{(\mu -{\eta }^{2})}{1-{\eta }^{2}},{\rm{B}}=\frac{{{\rm{\Omega }}}_{i}{{r}_{i}}^{2}(1-\mu )}{1-{\eta }^{2}}$$

At small angular velocities of the internal cylinder, the driven flow is laminar and purely azimuthal (the circular Couette flow, CCF). Taylor^15^ recognised that when the angular velocity of the internal cylinder exceeded a critical value, referred to by the critical Taylor number Ta, a primary instability developed whereby the flow became unstable to axisymmetric perturbations and the radial *v*_*r*_ and axial *v*_*z*_ velocity components manifest into steady stacked counter rotating vortices. This three-dimensional laminar flow is known as Taylor-Couette (TCF) flow and upon further increase of the rotation beyond a series of subsequent critical values, the flow becomes unstable to un-axisymmetric perturbations and azimuthal waves develop on the tori which eventually becomes chaotic leading to a fully turbulent regime^19^. The TCF problem has also been demonstrated for concentric cylinders with a free-surface^20,21^. Most notably from this, Dunst^21^ shows using observations of dye on a wide gap (small radius ratio) free-surface Couette flow, that the inertial stability of the flow depends largely on *μ* describing the momentum generation as a sink or a source. Using Rayleigh’s stability criterion^22^, Dunst^21^ highlighted that the whole flow field can become unstable with the rotation of only the inner cylinder. In such a case, Dunst^21^ also made observations of strong inwards radial flow at the base of the tank which became weaker in the sub-surface region. The inward flow for a Taylor-Couette flow was also noted by Ogawa^23^ prior to the formation of turbulence again on small radius ratio configuration. In general, these observations are similar to inward flow bands discussed by Anwar^10^ and Daggett and Keulegan^9^. In summary, many studies have been performed on this topic and the Taylor-Couette flow system has remained an ideal model to study instabilities, nonlinear behaviour and transitions to turbulence in fluid flows over the past number of decades^19,20,24–33^.

From both flow systems (the TCF and the FSV) it is interesting to point out that for the case of a fixed outer boundary, the velocity field decreases monotonically in a radial direction outwards^34^. In the following study, implementation of analytical, experimental and numerical analysis of the turbulent free-surface vortex chamber reveals a number of further compelling similarities between both flow systems. Most notably, experimental and numerical analysis confirms the existence of ‘Taylor-like’ vortices residing in the secondary flow field of the FSV which are proposed to be triggered by a centrifugal driving force. The formulated analogue between the TCF and the FSV systems differs mainly by energy transfer where the former is mechanical driven and the latter is driven by continuity of flow. However, in order to override this contrast, one simply has to visualise the air core of the FSV as a ‘virtual inner cylinder’ to perceive the reverse effect. We show that through this analogue, the instability mechanisms are indeed the same by considering Rayleigh’s stability criterion. As will be discussed, this discovery and descriptive analogue to the Taylor-Couette flow system has significant implications for a fuller understanding of turbulent free-surface vortex flow behaviour and stability.

## Results

### Instability mechanisms in strong free-surface vortices

Considering th vortex flow problem as outlined in (Fig. 1b) with no outer boundary. Using the quasi-cylindrical approach for an ideal fluid it is easy to infer from the radial momentum equation that *v*_*θ*(*FSV*)_ = *V*(*r*) = *Γ*_∞_/2*πr* as shown by others^34^ where *Γ*_∞_ is the constant circulation field. To compare generally with the circular-Couette flow (CCF), if we remove the external cylinder (or boundary) in this problem by setting *r*_*o*_ → ∞ and thus *η* → 0 in Equations (1) and (2), the tangential velocity profile then becomes ${v}_{\theta (CCF)}=V(r)=\,{{\rm{\Omega }}}_{i}{{r}_{i}}^{2}/r$, which is mathematically similar to the ideal free-surface vortex primary flow field. In order to extend this analogy, let us next consider the tangential momentum equation of the Navier-Stokes equations as follows:

3 $$\rho (V\cdot \nabla ){v}_{\theta }+\,\frac{\rho {v}_{r}{v}_{\theta }}{r}=\,-\frac{1}{r}\frac{\partial p}{\partial \theta }+\,\rho {g}_{\theta }+\,\mu ({\nabla }^{2}{v}_{\theta }-\frac{{v}_{\theta }}{{r}^{2}})$$

which, through the quasi-cylindrical approximation^3^, simplifies to:

4 $${\nabla }^{2}{v}_{\theta }=\,\frac{1}{r}\frac{d}{dr}(r\frac{d{v}_{\theta }}{dr})=\,\frac{{v}_{\theta }}{{r}^{2}}$$

This linear second order ordinary differential equation is then solved for the vortex flow system with an outer curved boundary to become:

5 $${v}_{\theta }(r)=\,A\text{'}r+\,\frac{B\text{'}}{r}$$

By isolating a radial section of secondary flow field and imposing the boundary conditions of rotation at the air core interface *r* = *r*_*a*_ (analogous to the rotating inner cylinder of the TCF) and at a stationary or non-stationary curved boundary some radial position *r* = *r*_*o*_ by *v*_*θ*_(*r* = *r*_*a*,*o*_) = *v*_*θa*,*o*_ = Γ_*r*_/*r*_*a*.*o*_, the equations for *A*′ and *B*′ become:

6 $$A^{\prime} =\frac{{v}_{\theta a}}{{r}_{a}}\frac{({\mu }_{v}-{{\eta }_{v}}^{2})}{1-{{\eta }_{v}}^{2}}\,{\rm{and}}\,B^{\prime} ={v}_{\theta a}{r}_{a}\frac{(1-{\mu }_{v})}{1-{{\eta }_{v}}^{2}}\,$$

where Γ_*r*_ is the circulation distribution along *r*, *μ*_*v*_ = *v*_*θo*_*r*_*a*_/*v*_*θa*_*r*_*o*_ and *η*_*v*_ = *r*_*a*_/*r*_*o*_. The free-surface vortex velocity field can then be solved to become:

7 $${v}_{\theta }(r)=\,\frac{{v}_{\theta a}}{{r}_{a}}\frac{({\mu }_{v}-{{\eta }_{v}}^{2})}{(1-{{\eta }_{v}}^{2})}r+\,\frac{{v}_{\theta a}{r}_{a}(1-{\mu }_{v})}{r(1-{{\eta }_{v}}^{2})}$$

Equation 7, for a stationary boundary *μ*_*v *_= 0 then becomes

8 $${v}_{\theta }(r)=\,-\frac{{v}_{\theta a}}{{r}_{a}}\frac{{{\eta }_{v}}^{2}}{(1-{{\eta }_{v}}^{2})}r+\,\frac{{v}_{\theta a}{r}_{a}}{r(1-{{\eta }_{v}}^{2})}$$

To investigate inertial stability in this case, consider two fluid particles of same mass positioned at *r* = *r*_1_ and *r* = *r*_2_ in the flow field as shown in Fig. 1f. The combined kinetic energy of both particles sums to:

9 $$E=\frac{1}{2}m(\frac{{{L}_{1}}^{2}}{{{r}_{1}}^{2}}+\frac{{{L}_{2}}^{2}}{{{r}_{2}}^{2}})\,$$

where *L*_1,2_ = *v*_*θ*1,2_*r*_1,2_ is the angular momentum. If under some circumstance or perturbation, both particles were to suddenly swap position, each conserves its angular momentum, and the new combined energy becomes:

10 $${E}_{new}=\frac{1}{2}m(\frac{{{L}_{2}}^{2}}{{{r}_{1}}^{2}}+\frac{{{L}_{1}}^{2}}{{{r}_{2}}^{2}})\,$$

Thus, the energy change from *E* → *E*_*new*_becomes:

11 $${\rm{\Delta }}E\propto ({{L}_{2}}^{2}-{{L}_{1}}^{2})(\frac{1}{{{r}_{1}}^{2}}-\frac{1}{{{r}_{2}}^{2}})\,$$

If the swap results in a release of energy $({\rm{\Delta }}E < 0,\,{{L}_{2}}^{2} < {{L}_{1}}^{2})$, the laminar flow will be unstable to such a perturbation. Thus the Rayleigh criterion^22^ for instability requires that the derivative with respect to *r* of the square of the angular momentum is less than zero by

12 $$\frac{d}{dr}{({v}_{\theta }r)}^{2} < 0$$

By substituting Equation 8 into Equation 12 it is possible to show that the secondary flow field of the FSV with an outer stationary wall can become unstable to axisymmetric perturbations in an analogous manner to the TCF.

**Figure 2:**
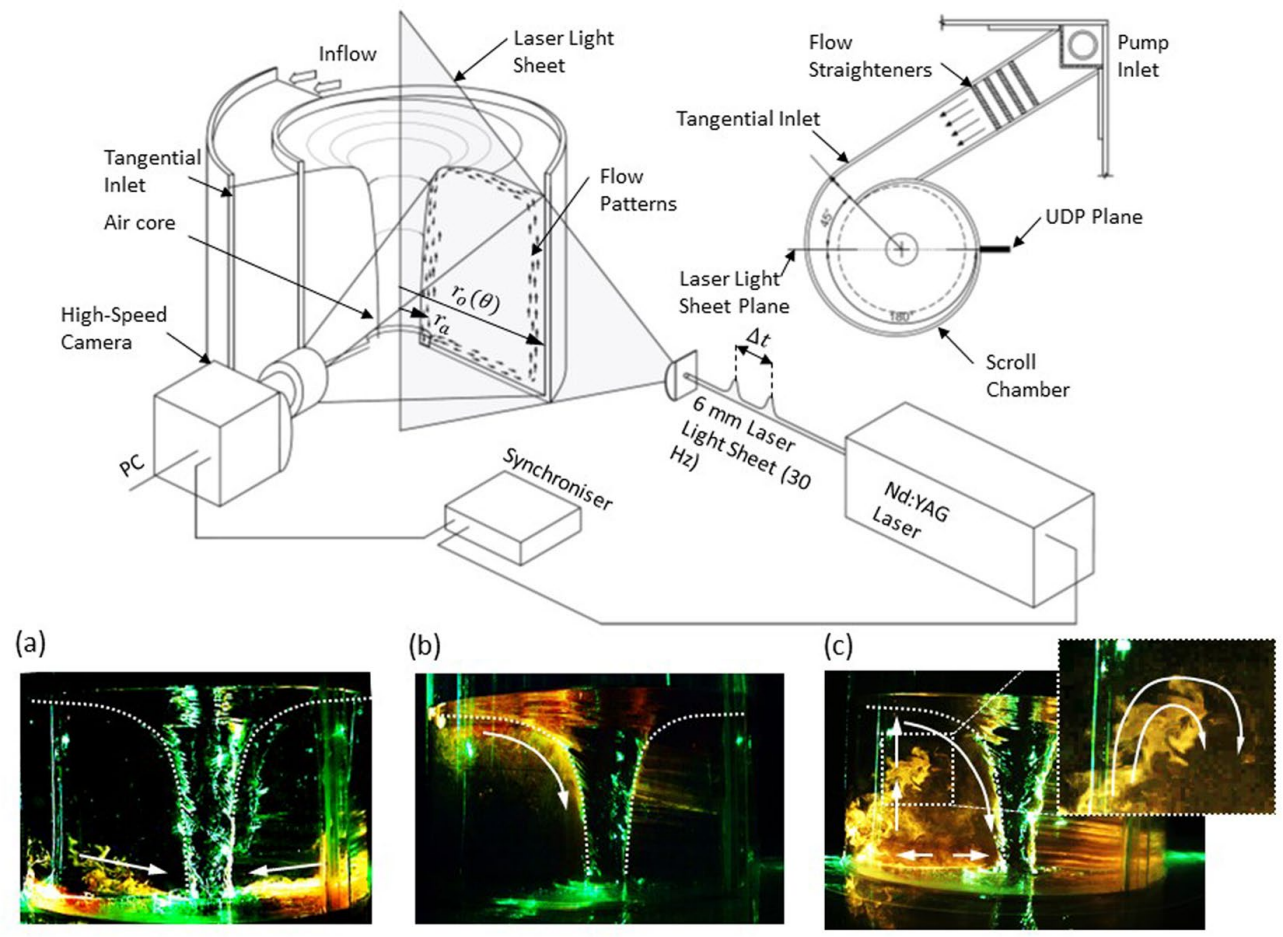
The planar laser induced fluorescence (PLIF) technique was used to provide a visualisation of the secondary flow field patterns. Rhodamine B dye was injected at (**a**) the vessel base and (**b**) the free-surface close to the inlet channel and (**c**) at the chamber perimeter which *r*_*o*_(*θ*). The presented images had a vortex Reynolds number of approximately Re_Γ_ = 1.7 × 10^5^ and M = 6.4 × 10^5^. The dye was observed to be confined to bands along the tank base and at the free-surface in an inward positive direction which were in line with the observations of Anwar^10^ and Daggett and Keulegan^9^. A new flow process was also observed where the dye travelled upwards along the tank perimeter to convey a secondary flow to the free-surface and downwards at the near the vortex core at radius *r*_*a*_. This supplementary process outlined that some separation zone, possibly transient in nature, must occur at the tank base or walls. The flow processes outlined that the flow in global secondary field was quasi-toroidal. Figure 2c also highlights that the flow field exhibited some evidence of rotation.

For the purpose of investigating the aforementioned analogue further, a strong and steady free-surface vortex flow, using water as the fluid, is generated in a scroll type vortex chamber where the walls follow a logarithmic spiral *r*_*p*_(*θ*) = *ae*^*bθ*^ centred about a 67 mm discharge as outlined in Fig. 2. A flow *Q* generated by pumping is conveyed into a baffled channel which creates a steady hydrostatic approach flow depth *h* in the tangential inlet. Six subcritical steady state approach flow depths were examined (1.38 × 10^5^ ≥ Re_Γ_ ≥ 2.07 × 10^5^). A full description of the experimental test rig is available in the following methodology section and also in a study performed by Mulligan *et al*.^35^.

To facilitate a comparison with the Taylor-Couette flow, the variation of vortex flow depth *h* (to vary the energy of the flow system) ensued various aspect ratios defined by *ζ*_*v*_ = *h*/(*r*_*o*_(*θ*) − *r*_*a*_). The radius ratio also varied with respect to *θ* and *r*_*a*_ by *η*_*v*_(*θ*, *r*_*a*_) = *r*_*a*_/*ae*^*bθ*^. Thus the geometry, and any geometry induced instability mechanisms, must be asymmetric (effects of which will be discussed later). In order to isolate the dependence of the spiral walls, measurements and observations were undertaken at a fixed secondary plane of *θ* = 45 ° and 225 ° for planar laser induced fluorescence observations and ultrasound Doppler profiling as outlined in Fig. 2 where the inlet is positioned at *θ* = 0°. To be consistent with the TCF, we also formulate a new variation of the vortex Reynolds number Re_Γ_ to take into account the characteristic radii defined by the gap between the air core and the outer most boundary (*r*_*o*_ − *r*_*a*_) by:

13 $${\rm{M}}=\frac{{{v}_{a}}^{2}}{{r}_{a}}\frac{{({r}_{o}-{r}_{a})}^{3}}{{\nu }^{2}}=\frac{{\Gamma }_{r}}{2\pi {{r}_{a}}^{\frac{3}{2}}}\frac{{({r}_{o}-{r}_{a})}^{\frac{3}{2}}}{\nu }={\bf{R}}{{\bf{e}}}_{{\rm{\Gamma }}a}\,\frac{{({r}_{o}-{r}_{a})}^{\frac{3}{2}}}{2\pi {{r}_{a}}^{\frac{3}{2}}}$$

### Global secondary flow field behaviour

Initially, qualitative secondary flow field observations were made using the elements of PLIF (Fig. 2) to provide basic dye advection observations. Figure 2a and b indicates the dominant flow patterns observed in the secondary flow field using PLIF. By injecting a small quantity of Rhodamine B dye at the tank base and at the free-surface, advection layers were observed to develop in both regions which demonstrated radial inflow towards the orifice and downwards axial flow at the vortex core. The results were in line with the observations made by^9,10^. Additional transport of the dye was observed in an upward axial direction along the chamber walls which conveyed flow to the free-surface and consequently the core region. This revealed a global quasi-toroidal flow pattern in the secondary flow field. The system was entitled ‘quasi-toroidal’ owing to the fact that the toroidal loop was strictly unclosed due to a transient separation point occurring at some region near the floor or wall as shown in Fig. 2c.

### Radial and axial velocity profiles using UDP

**Figure 3:**
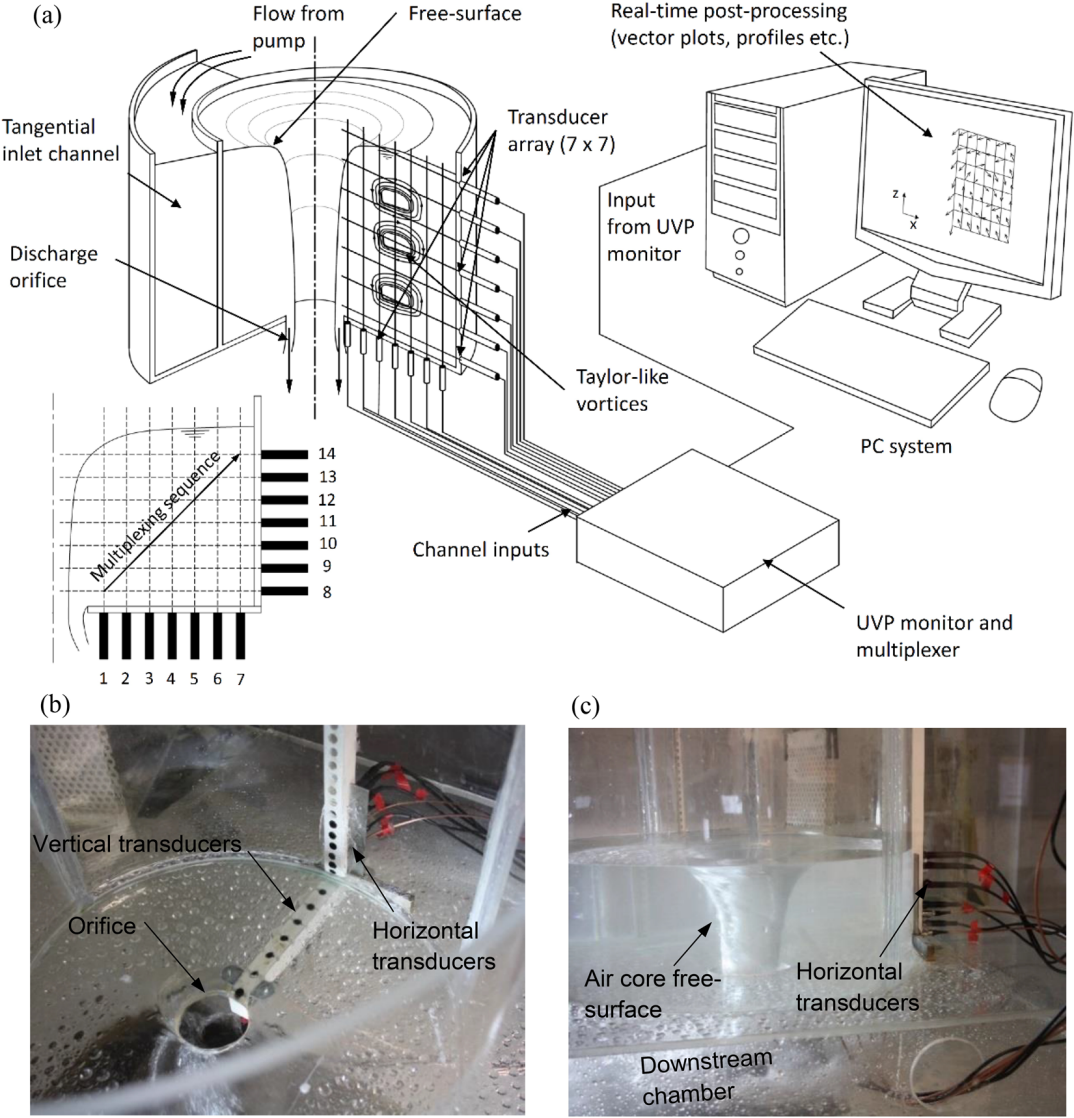
2D ultrasound Doppler profiling (UDP) flow mapping technique utilising a 7 × 7 array aligned with the *r*–*z* axis passing through a semi-cross section of the vortex core as outlined in (**a**) 3D schematic of overall testing configuration together with (**b**) an image of the ultrasound transducers installed on the vortex chamber and (**c**) image of transverse transducers and seeded vortex flow during testing. The transducers were spaced at 22.5 mm centres along the horizontal (*r*–axis vertical transducers) and 12.5 mm centres along the vertical (*z*–axis horizontal transducers) as highlighted. The first transducer on the vertical and horizontal were placed at 15 mm and 13 mm away from the boundaries respectively. The transducers were triggered diagonally in sequenced pairs (i.e. 1 & 8, 2 & 9 and so on) in order to detect high velocity gradients in the near-field core regions earlier in the sweep. The geometric values for the chamber are outlet size *d* = 0.067 m, inlet width *b* = 0.067 m and inlet radius of *r*_*in*_ = 0.207 m corresponding to an approach flow geometric factor *α* = 3.129.

In order to provide a detailed quantitative examination of the global secondary flow field at 225 °, velocimetry was performed using UDP and an original flow mapping technique which was a first application of 2D array UDP in free-surface draining vortices. High spatial and temporal resolution *v*_*r*_ and *v*_*z*_ profiles at various *z* and *r* positions respectively was achieved using the array configuration of UDP transducers outlined in Fig. 3, which leveraged the approaches of others on TC systems^19,36,37^. Individual profiles were obtained at 19 ms with an in axis resolution of resolution of 2.5 mm/s. This arrangement also permitted 2D velocity flow maps to be constructed over the secondary flow field. Further information on the UDP configuration is outlined in the following methods section.

**Figure 4:**
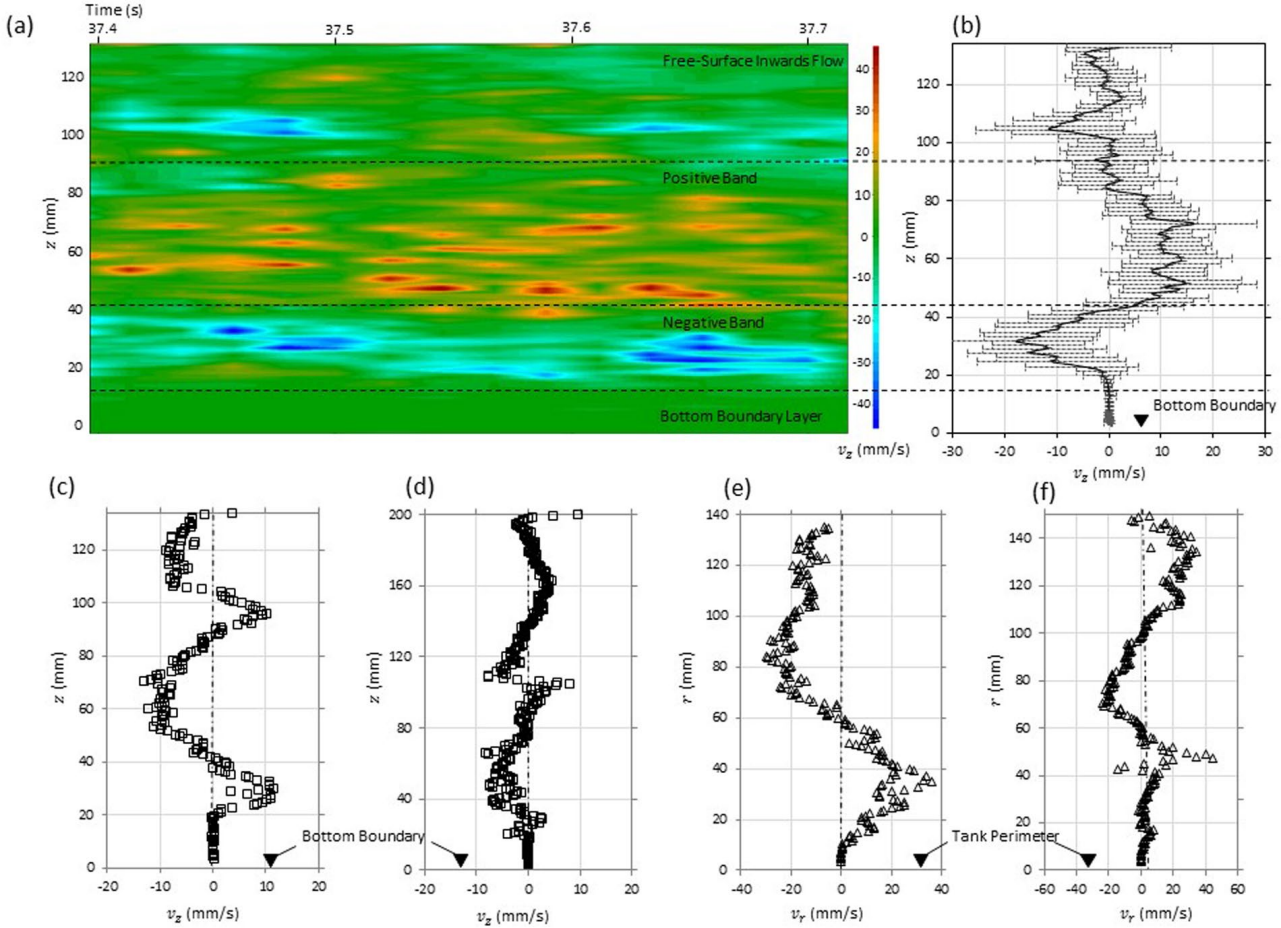
Spatio-temporal map representing the magnitude of the axial velocity along the *z*–axis for a approximately 0.3 seconds is highlighted in (**a**) which was achieved using (**b**) transducer 4 positioned at 80.5 mm away from the vessel perimeter. Based on observations of the velocity vector maps, the vertical transducer 4 provided a profile which was likely to pass through the regions of Taylor-like vortices. The counter rotating Taylor-like vortices are represented on the (**a**) spatio-temporal colour map by the negative, positive and negative radial bands which appear to span the extent of the *z*–axis indicating areas of alternating rotation; a signature of the Taylor vortices. Figure 4(c,d) Presents the axial velocity profiles obtained using transducer 4 for *h*/*d* = 1.5 (**M** = 6.37 × 10^5^) and *h*/*d* = 2.0 (**M** = 7.42 × 10^5^) which highlight the signature for cellular structures in the secondary flow field. The radial velocity *v*_*r*_ along the radius determined from transducer 8, positioned at 15 mm above the base, is highlighted for (**e**) *h*/*d* = 1.5 (**M** = 6.37 × 10^5^) and (**f**) *h*/*d* = 2.0 (**M** = 7.42 × 10^5^) highlighting inwards and outwards radial flow zones.

Figure 4a displays the quantitative signature of the cellular structures using a spatio-temporal map of the axial velocity distribution for *h*/*d* = 2.0 where the axial velocity alternates between the negative downward and upward positive profiles passing through counter rotating zones. The spatio-temporal map indicates that the vortices do not vary significantly within the 0.3 second time frame as indicated by the approximate positive and negative bands determined along the transducer 4 profile (Fig. 3b). Figure 4b highlights the standard deviation of the average velocity profile determined throughout the 0.3 s interval consisting of 16 profiles (19 ms sampling period).

**Figure 5:**
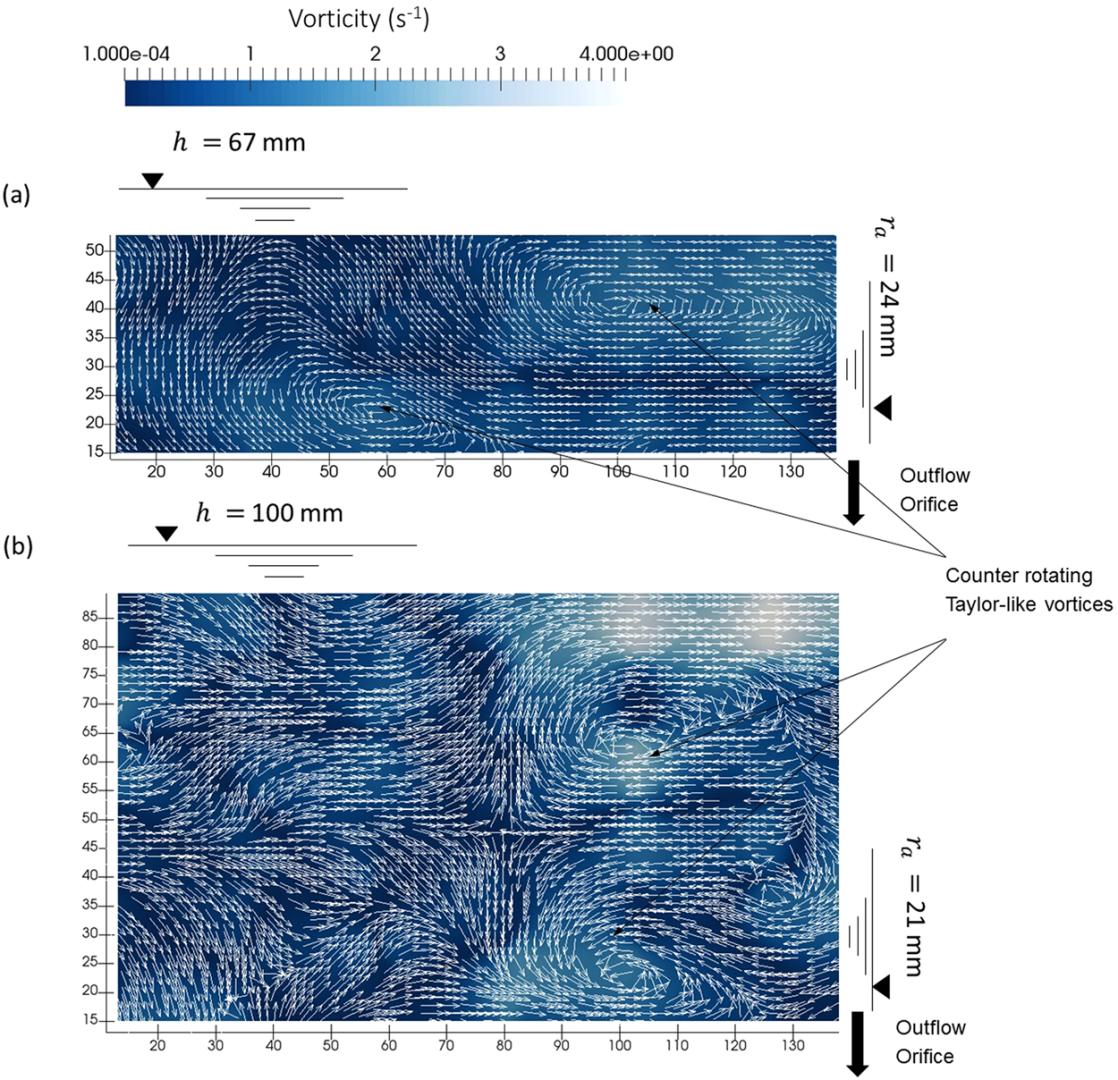
2D velocity vector fields (*v*_*r*_ and *v*_*z*_) determined from UDP flow mapping process for two free-surface vortex flows. (**a**) *h*/*d* = 1.0, *Q* = 0.635 l/s, **N**_Γ_ = 16.83, *a*_*c*_/*d* = 0.739, **M** = 4.76 × 10^5^, *η*_*v*_ = 0.127 and *ζ*_*v*_ = 0.4 and (**b**) *h*/*d* = 1.5, *Q* = 1.1 l/s, **N**_Γ_ = 10.4, *a*_*c*_/*d* = 0.649, **M** = 6.37 × 10^5^, *η*_*v*_ = 0.11 and *ζ*_*v*_ = 0.58. Each plot displays the velocity vectors and absolute vorticity contours together indicating the positions and magnitudes of observed ‘Taylor-like’ vortices together with the approximate position of the free-surface profile.

Figure 4c,d,e and f highlights instantaneous axial (transducer 4) and radial (transducer 8) profiles for *h*/*d* = 1.5 and 2.0 respectively. Figure 5e and f indicates that there is an outward flow near the tank periphery in both cases whereas near the core the flow can switch between inward (negative velocity) and outward (positive velocity) flows. The vertical position of the horizontal transducer 8 at 11 mm above the base is such that the beam profile is not completely within the bottom flow band outlined in the PLIF experiments. Hence, near the core at a short distance up from the base of approximately 10 to 30 mm there is highlight unsteady, oscillating behaviour. This inward, outward switching of radial velocities developing close to the air core for high depths *h*/*d* 1.5 was in line with the observations made by in Fig. 4 and by Anwar^10^ on small suspended particles in the flow field in this region.

For low approach flow depths of *h*/*d* ≤ 1, the radial and axial velocity did not reveal cellular flow structures and only highlighted flows residing at the boundaries. Any observations of the flow field above an approach flow depth of *h*/*d* = 1.5 (M = 2.22 × 10^5^ and *ζ*_*v*_ = 0.58) exhibited definite unsteady characteristics in the secondary flow field; i.e. the position and magnitude of the vortices varied with time. A prolonged time series for the axial velocity at a sub-surface point in space indicated that the flow field was quasi-periodic or chaotically wavy, similar to that described by Takeda^19^ in Taylor-Couette flows. Both the core tangential and axial velocities were recorded to be of the order of 1 to 2 m/s whereas, in all cases, the Taylor-like vortices exhibited velocities that were two orders of magnitude smaller than the core velocities at 0.005 to 0.05 m/s. This is very similar to the observations made by Dunst^21^ on a free-surface Taylor-Couette flow with a small radius ratio. Dunst^21^ also found a strong inward flow near the bottom and a weak outward flow in the sub-surface region indicating that there is a ‘source’ of angular momentum generated. The transition between the inward flow band and the weak outward flow is analogous to the observations made in this region in the FSV using UDP.

### Taylor-like vortices

Due to the relative stability of the cellular structures observed in Fig. 4a and b, it was possible to use the transducer array to construct quasi-instantaneous 2D velocity vector maps for various *h*/*d*. As shown in Fig. 5a and b, the 2D flow maps confirmed the existence of ‘Taylor-like’ vortices where loosely structured counter rotating cellular structures can be observed. The loosely structured features may have been a result of the small radius ratio 0.152 ≥ *η*_*v*_ ≥ 0.090 resulting in a lower instability threshold together with the large Reynolds number in the flow field of the order of 10^5^. Residual artificial perturbations introduced from the inlet conditions may also have contributed to their loosely structured appearance. No cells were observed for *h*/*d* = 0.5 (*ζ*_*v*_ = 0.2); one or two cells were observed when *h*/*d* ≥ 1.0 (*ζ*_*v*_ ≥ 0.4) and two or three cells were observable when *h*/*d* ≥ 3.0 (*ζ*_*v*_ ≥ 1.0) which describes that the number of cells increase with aspect ratio *ζ*_*v*_ similar to Watanabe and Toya’s^20^ observations on small aspect ratio free-surface TCFs.

**Figure 6:**
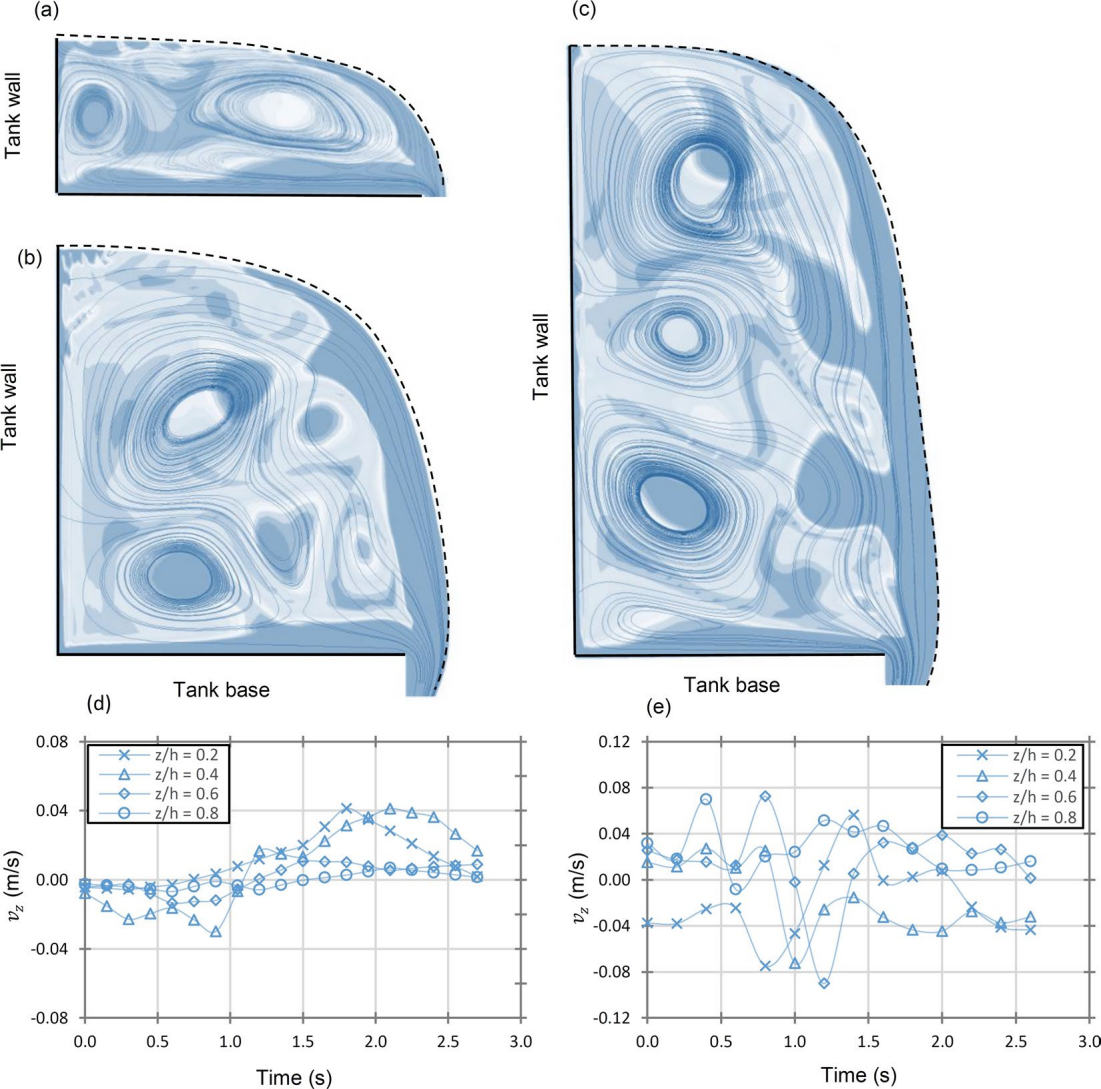
Results of three-dimensional multiphase models of the free-surface vortex using the Reynolds stress turbulence modelling is presented for (**a**) *h*/*d* = 1.0, *Q* = 0.70 l/s, **N**_Γ_ = 12.77, *a*_*c*_/*d* = 0.75, **M** = 3.92 × 10^5^, *η*_*v*_ = 0.158 and *ζ*_*v*_ = 0.50 (**b**) *h*/*d* = 2.0, *Q* = 1.70 l/s, **N**_Γ_ = 6.38, *a*_*c*_/*d* = 0.51, **M** = 9.33 × 10^5^, *η*_*v*_ = 0.107 and *ζ*_*v*_ = 0.95 and (**c**) *h*/*d* = 3.5, *Q *= 3.10 l/s, **N**_Γ _= 3.73, *a*_*c*_/*d* = 0.44, **M** = 1.26 × 10^6^, *η*_*v*_ = 0.093 and *ζ*_*v*_ = 1.64 for a semi-cross section of the vortex chamber at *θ* = 45 ° using instantaneous absolute vorticity contour plots and streamlines. The position of the free-surface determined using the volume of fluid method is provided using an iso-surface positioned at a volume fraction *φ* = 0.5. The secondary flow fields displayed the presence of Taylor-like vortices in each case which is in agreement qualitatively with the observation of the UDP results. The secondary flow field was steady for low approach flow depths (*h*/*d* = 1.0, **M** = 3.92 × 10^5^) but became unsteady as the approach flow depth increased beyond M = 9.33 × 10^5^. Figure 6(d) presents the transient evolution of various points along the z axis (*z*/*h*) at 0.1 m from the vortex centre. A chaotic wavy pattern emerged for the (**e**) *h*/*d* = 3.5 indicating the onset of turbulence as the approach flow depth increased. The geometric values for the chamber are outlet size *d* = 0.067 m, inlet width *b* = 0.067 m and inlet radius of *r*_*in*_ = 0.174 m corresponding to an approach flow geometric factor *α* = 2.59.

A final clarification of the composition of the secondary flow field was achieved through three-dimensional transient numerical modelling using Reynolds stress turbulence modelling. The simulation was performed on a model with a larger radius ratio 0.09 ≥ *η*_*v*_ ≥ 0.16 for approach flows of *h*/*d* = 1.0, 2.5 and 3.5. Figure 6a–c presents instantaneous streamlines and vorticity contours generated on the secondary flow field. Similar to Figs 2 and 4, the secondary flow field was found to be composed of strong advection layers forming at the base of the tank, the free-surface, vessel walls and orifice with superimposed cellular structures. The cellular structures originated at the tangential inlet once the fluid underwent streamline curvature and appeared to wrap and spiral around the vortex core terminating close to the bottom outlet. For the low approach flow depths of *h*/*d* = 1.0 the flow field appeared to retain a rotational three-dimensional laminar flow which often bifurcated to two parallel cells (Fig. 6a) before merging again into a single cell. At *h*/*d* = 2.0 (*ζ*_*v*_ = 0.95), the flow field comprised of two well-structured stable cells as shown in Fig. 6b. At *h*/*d* = 3.5 (*ζ*_*v*_ = 1.637) three cells were apparent in the flow field and the Taylor-vortices exhibited wavy patterns. Figure 6e and f highlights the variation of the *v*_*z*_ with time *t* at various axial positions *z*/*h* along the radial position *r* = 0.1 m. As the approach flow depth is increased from *h*/*d* = 1.5 to 3.5, the *v*_*z*_ time series varies from a lightly transient (wavy) (Fig. 6e) to a state to highly transient chaotic state (Fig. 6f).

## Discussion

We have shown that strong free-surface vortices exhibit strong similarities to the Taylor-Couette flow system through the flow mechanics of the primary and secondary flow field and observations of “Taylor-like” vortices. The various levels of unsteadiness could be lightly classified into various modes of instability similar to the TC flow up to the chaotic wavy mode. However, the exact instability thresholds of each qualitatively observed feature is still up for question. Table 1 provides a summary of the chief analogous features between both flow systems as derived from and observed within this study.

**Table 1:** The analogue between the Taylor-Couette Flow and the Free-Surface Vortex.

**Feature**	**Taylor**-**Couette** (**TC**) **Flow**	**Free**-**Surface Vortex Flow**
*Coordinates*	*r*, *θ*, *z*	*r*, *θ*, *z*
*Energy induction*	Mechanical rotation of cylinders which imparts rotation Ω_*i*_ through fluid shearing	Continuity of mass flow in and out of the system driven by gravity *g* which imparts global field circulation Γ∞
*Global flow behaviour*	Flow in and out of the system is zero.	Flow in and out of the system is non-zero
	*Q* = 0	*Q* ≠ 0
	Volumetric flux is zero but fluid moves concentrically in the system	Definite volumetric flux ensured by radial and axial flow
*Conservation of energy*	The energy loss in the domain generated by viscous friction is balanced by mechanical energy input Ω_*i*_ (shear driven flow)	Energy loss in the domain through viscous friction but replenished or balanced by new incoming flow driving circulation Γ∞ (shear driven flow)
*Unstable driving force*	Centrifugal force ${F}_{g}\propto m{{{\rm{\Omega }}}_{i}}^{2}{r}_{i}$	Centrifugal force ${F}_{g}\propto \,m\frac{{{v}_{\theta a}}^{2}}{{r}_{a}}$
*Primary velocity field*	*v*_*θ*_ = *V*(*r*) = *Ar* + (*B*)/(*r*)	*vθ* = *V*(*r*) = *A*′*r* + (*B*′)/(*r*)
	where	Where
	$A=\,{{\rm{\Omega }}}_{i}\frac{(\mu -{\eta }^{2})}{1-{\eta }^{2}},\,{\rm{B}}=\frac{{{\rm{\Omega }}}_{i}{{r}_{i}}^{2}(1-\mu )}{1-{\eta }^{2}}$	$A^{\prime} =\,\frac{{v}_{\theta a}}{{r}_{a}}\frac{({\mu }_{v}-{{\eta }_{v}}^{2})}{1-{{\eta }_{v}}^{2}},\,B\text{'}={v}_{\theta a}{r}_{a}\,\frac{(1-{\mu }_{v})}{1-{{\eta }_{v}}^{2}}$
*Dimensionless driving control parameter*	${\rm{Ta}}\,=\,\frac{{r}_{i}{{{\rm{\Omega }}}_{i}}^{2}{({r}_{o}-{r}_{i})}^{3}}{{\nu }^{2}}$	${\rm{M}}\,=\,\frac{{{v}_{a}}^{2}}{{r}_{a}}\frac{{({r}_{o}-{r}_{a})}^{3}}{{\nu }^{2}}$
*Secondary flow field*	Comprised of well-structured and stacked counter rotating Taylor-vortices	Comprised of loosely structured counter rotating ‘Taylor-like’ vortices superimposed on a global quasi-toroidal flow around the edges of the domain
*Steadiness*	Taylor-vortices remain steady and axisymmetric for low Ω_*i*_ and low Ta	‘Taylor-like’ vortices are asymmetric and remain steady for low *Q* and low M
*Unsteady characteristics*	Progresses to wavy, quasi-periodic wavy, fast azimuthal wave, soft turbulence, hard turbulence and complete turbulence as Ta progresses beyond a series of critical values	Progresses to unsteady, wavy, quasi-periodic wavy and turbulent instability modes as M progresses beyond a series of critical values. More discrete descriptions have yet to be identified.

The primary difference between the free-surface vortex and Taylor-Couette flow lies in the process of energy transfer. In the TCF, mechanical energy is introduced by the rotating inner and outer cylinders that drive the flow via shear and momentum transfer between fluid layers. In major contrast to this, the free-surface vortex flow system behaves in reverse, where the central core derives rotational energy from shear driven circulation field derived from a continuity of flow entering the domain as described by Anwar^10^ and Daggett and Keulegan^9^. In order to formulate a more complete description, developing on studies from others^9,10^ we propose that it is the global quasi-toroidal flow process in the secondary flow field that provides the discharge, and thus, the energy to maintain the vortex (i.e. to bring in new energy to replace that lost to viscosity or turbulence). Thus, this energy from a continuity of flow is analogous to energy introduced by mechanical rotation in the TCF. This hypothesis has significant implications in developing a more unified understanding of the dynamics of the secondary flow field in both strong and weak turbulent free-surface vortex flows as is discussed next.

Despite the dissimilarity in the energy induction process, the free-surface vortex can be treated analogously as a Taylor-Couette flow by visualising the vortex air core as a ‘virtual cylinder’. That is to say, for this analogy to be valid we are not obliged to visualise an air core, and similarly the fluid flowing inwards at the boundaries may be supposed to be removed, as its energy maintains the rotation of the ‘virtual inner cylinder’. The analogue was coined in this study as the ‘Analogous Taylor-Couette’ flow insofar that, as the energy is increased due to inflow in the free-surface vortex, the air core (or virtual cylinder) narrows and increases in velocity resulting in the generation of rotational instabilities in the secondary flow field in an analogous manner to the Taylor-Couette system. The unstable driving force in both flow systems is then a centrifugal one which is to be balanced by the pressure gradient for the flow to remain stable. Thus in the free-surface vortex, the secondary flow field primary instability is developed when a fluid particle is perturbed from its initial position in, say an outward radial direction, beyond the point where the local restoring force due to the pressure gradient is slightly less than the outward inertia of the particle, resulting in the particle continuing to move outwards. *Vice versa*, a fluid particle perturbed inward will continue inwards and mass conservation will ensure toroidal cell development as shown in Fig. 1f.

However, according to the Rayleigh stability criteria, the secondary flow field can only become unstable when it is contained between an outer wall. As applied to the unbounded case of Fig. 1b where *r*_*o*_ → ∞ and *η*_*v*_ → 0(i.e. the case of infinite curvature) it is easy to deduce from Equations 7 and 8 that the primary flow field is fully irrotational and the flow is stable. The same effect can theoretically occur when you have a bounded free-surface vortex flow where the air core radius *r*_*a*_ → 0 (i.e. the vortex flow approaches the critical submergence depth *S*_*c*_). However, since azimuthal shear instabilities (which occur for high core velocities) are also associated with a decrease in azimuthal circulation, centrifugal instabilities are likely to render the flow unstable in each case similar to the Taylor-Couette flow^38^. Furthermore, for weak vortices when *h* ≥ *S*_*c*_, Gallaire and Chomaz^38^ describe how a centrifugal instability can occur across the Rankine vortex tangential velocity distribution. It is also important to point out that the strong axial downward flow at the core, combined with the fixed boundaries of the base and wall, also renders the problem analogous to a cavity driven flow^39^. Therefore, instabilities due to axial motion have also to be considered to evaluate the nature of three-dimensional, anisotropic turbulence also including azimuthal shear instabilities^40^. As discussed previously, although the primary flow field is taken to be largely axisymmetric, the secondary vortex instabilities are likely to be asymmetric due to the asymmetry of the approach flow geometry in the current case (*r*_*o*_ ≥ *ae*^*bθ*^), thus, the critical instability threshold varies as one circumferentially spans the system. It is also worth noting that the current analogue may be extended other variations of the TCF system which introduce axial effects (i.e. Spiral—Couette and Spiral—Poiseuille flows^40^) which may also help describe the formation of asymmetric Taylor-like vortices. The extension of the analogue to consider Spiral—Couette and Spiral—Poiseuille flows^40^ would no doubt be an interesting study.

In more general terms, it is hypothesised that the primary instability of “Taylor-like” vortex behaviour aids the flow system in avoiding chaos in the distribution of flow and energy from the inlet to the outlet regions of the domain while maintaining primary field rotation and an ‘open vortex’. This is inferred from the streamlines originating near the cells shown in Fig. 6 which appear to supply flow to the boundaries and subsequently to the outlet. The Taylor-like secondary vortices were observed to asymmetrically ‘wrap’ around the primary vortex core (or primary vortex line).In general, the cellular wrapping behaviour is close in appearance to the spatiotemporal processes described by Chanaud^41^ on observations of oscillatory motion in the breakdown of swirling flows. Given this, it is well-known that as the approach flow depth (or system energy) is increased beyond the critical depth, the vortex collapses to form a dimple and the velocity distribution follows a Rankine distribution^1,7^. To the best of our knowledge, there has been no fluid mechanical description for the role of the secondary flow field during this critical transition, nor a description for the decay according to a Rankine distribution besides the fact that turbulence plays a significant role^34,42^. A possible explanation may lie in the concept of the Taylor-Couette analogy where, as the modes transition from a chaotic wavy to a turbulent flow regime similar to the turbulent Taylor-vortices^19,43^, the combination of centrifugal axial and shear instabilities may, in turn, lead to breakdown and highly anisotropic turbulent flow conditions. There is also a nexus between this line of thinking and the vortex eddy viscosity concept introduced by Anwar^10^ and Einstein and Li^34^. In these articles, the authors discuss vortex stability on the basis of turbulent momentum transfer without providing a complete description of the origin of this turbulence.

Describing dynamic systems analogously is not a new topic in fluid and particle mechanics. A classic example is the strong similarity between the Taylor-Couette flow and the Rayleigh-Benard Convection^17^ system first noted by Rayleigh where he published work on the dynamics of rotating fluids^22^ and convection^44^. The analogy between the stability of rotating flows and the stability of stratified flows was also analysed by others^17,45–48^. Strong analogues have also been identified in an interdisciplinary context where Conway *et al*.^49^ demonstrated experimentally that granular material flows generate vortices consistent with the Taylor-Couette flow in fluids.

Much of meteorology^50^ and astronomy^31^ depends ultimately upon the dynamics of revolving fluids. As stated by Rayleigh, it is therefore desirable to formulate conclusions from “*simple approaches within our reach in the hope that they assist our judgement when an exact analysis or observation is impractical*”^22^. For that reason, many studies^51,52^ have considered the Taylor-Couette flow as a model to extend to complex astrophysical processes. For example, the instability of viscoelastic Taylor-Couette flow was found to be directly analogous to the magnetorotational instability in astrophysical magnetohydrodynamics by Ogilvie and Potter^53^ and further studied by Altmeyer *et al*.^54^. Perhaps, free-surface vortices, due to their gravitational inflow and outflow properties (absent in the Taylor-Couette flow) together with secondary flow rotational instabilities, may provide further insight into the behaviour of large meteorological systems^16,54–56^ or flows on a grander cosmological scale such as black hole accretion disk dynamics^57^. Jones^58^ speculates that the galactic structure may be maintained by an inflow of stars and gas as they fall into the black hole; directly analogous with how a free-surface vortex is maintained^9,10^. This would not be the first time that ‘kitchen sink cosmology’ is considered to offer a model for astrophysical systems on a table-top scale^58–60^. A well-planned Analogous Taylor-Couette system, with consideration for a proper fluid medium may provide an insightful system representation.

Currently, the article gives rise to many unanswered questions such as the magnitude of the critical thresholds, the effects of symmetric boundaries, varying gap width and the effects at the critical submergence. To fully realise the potential of the Analogous Taylor-Couette approach, it will certainly be necessary to perform careful systematic experimental studies on the secondary flow field to determine various values of the M parameter through various geometries, particularly at the onset of the critical submergence. We hope to contribute significantly to progress in this regard in future publications.

## Methods

### Hydraulic test rig and physical models

Details of the test configuration used in this article are presented schematically in the study of Mulligan *et al*.^35^. The physical model employed in the experimental study was constructed from 6 mm transparent acrylic and had an orifice diameter of *d* = 0.067 m, inlet width of *b* = 0.067 m and an inlet radius of *r*_*in*_ = 0.207 m corresponding to an approach flow geometric factor *α* = 3.129. The walls scrolled inwards according to the logarithmic spiral *r*_*p*_(*θ*) = 0.24*e*^−0.057θ^. The test geometries were mounted in a 0.85 × 0.95 m tank which was 0.5 m deep. The tank contained a centrally positioned 0.1 m orifice and rested on a platform over the storage reservoir. A 0.15 m high chamber was used to allow a void to permit the free discharge of flow from the orifice and to give ample space for UDP measurement on the tank underside. The model discharged the test flow to the drop shaft which transferred the flow to the lower storage tank.

The inlet to the test models comprised a 0.065 m bell mouth pipe entrance, 0.14 m perforated plates and honeycomb flow straighteners which homogenised the incoming velocity profile. Water was circulated through the system by a centrifugal pump (flow rate of 0 to 3.5 l/s) and was monitored using a magnetic flow meter (*B*-*Meters*, Italy) and regulated using two valves. The system monitoring delivered an accuracy corresponding to maximum error bars for intake Froude and radial Reynolds numbers of Fd = ± 0.019 and Rr = ±34, respectively. Approach flow depths *h* were expressed as a ratio of the constant orifice diameter *d* = 67 mm by *h*/*d*. Tests were performed for 6 approach flow depths corresponding to *h*/*d* = 0.5, 1.0, 1.5, 2.0, 2.5 and 3.0. This resulted in an experimental range of vortex Reynolds number Re_Γ_ = 1.38 × 10^5^ to 2.07 × 10^5^ and circulation numbers N_Γ_ = 5.82 to 31.03.

### Laser induced fluorescence

A visual observation of the global secondary flow field was achieved using planar laser-induced fluorescence (PLIF). PLIF is an optical flow visualisation technique used to observe the advection and diffusion of a fluorescent dye in the flow. The PLIF process is outlined in Fig. 2. In this application, a 532 nm Nd:YAG laser (*Spectron* SL404) was used in conjunction with Rhodamine B fluorescein which fluoresces at a wavelength λ = 590 nm wavelength (yellow) under the absorption λ = 532 nm light. The laser emits a 6 mm diameter beam profile which was passed through light sheet optics to generate a 2 mm thick, vertically orientated diverging laser light sheet which was directed to highlight the *z* − *r* plane residing at the centre of the vortex. A small quantity of Rhodamine dye was injected at (a) the tank base and at (b) the free-surface at the inlet channel and (c) at the chamber walls and a high-speed camera, positioned perpendicular to the light sheet arrangement, observed the advection mechanisms of the dye. The light sheet and transport of the fluorescein in each observation was imaged at a fixed pulse repetition and frame rate of 30 Hz.

### Ultrasonic doppler profiling

An original application of the Ultrasound Doppler Velocity Profile method (UDP method) was developed for analysing the secondary flow field in the free-surface vortex. The UDP principle utilises both echography and the Doppler Effect to respectively determine the position and velocity of a particle along an ultrasonic beam profile and is described in detail by the pioneer of the method, Takeda^36,37,61^. In our approach, we adopted a two-dimensional (2D) flow mapping principle by establishing a 7 × 7 array of ultrasonic transducers along the *r* − *z* plane of the vortex to ascertain 2D velocity vectors for a semi-cross section of the secondary flow field. The transducers could be placed at minimum and maximum centres of 25 mm along the horizontal (*r* − axis vertical transducers) and 22 mm along the vertical (*z*–axis horizontal transducers) as highlighted in Fig. 3. A total of 277 samples (channels) were achieved along each profile. A transmitting frequency of 4 MHz was used together with 4 cycles per pulse resulting in a minimum measurable channel width of 0.74 mm and the distance between channels was 0.74 mm. A pulse repetition frequency of 3.52 kHz was used resulting in an in-axis velocity resolution of 2.545 mm/s. Due to an ample concentration of seed particles available in the fluid (10 micron hollow glass spheres), a number of repetitions N_rep_ = 32 sufficed resulting in a time resolution of 9 ms. The transducers were multiplexed in a diagonal fashion using the following trigger sequence with reference to Fig. 3 (*S*_*t*_ = 1–8, 2–9, 3–10, 4–11, 5–12, 6–13, 7–14). 40 flow maps were obtained for each test over a duration of 224 seconds. The 2D velocity fields were smoothed using kriging interpolation and post processed using *ParaView*. The UDP system was supplied by *MetFlow SA* (Lausanne, Switzerland).

### Three-dimensional numerical modelling using multiphase reynolds stress modelling

Numerical modelling was performed using the commercial CFD software *ANSYS CFX* (V14.5) which uses a hybrid finite-element/finite-volume (finite element based finite volume method) approach to discretising the Navier-Stokes equations. Global conservation is satisfied by enforcing local conservation over the control volumes. The finite element approach is used to determine various surface fluxes and source terms within each element. Advection fluxes are evaluated using a high-resolution scheme that is second-order accurate and bounded. The two phase fluid domain was modelled using a homogeneous Eulerian-Eulerian multiphase flow model assuming that interphase momentum transfer is negligible. This was valid for the current test case where the phases were completely stratified and the interface was well defined. In the homogenous approach, both phases are treated as interpenetrating continua parted by a well-defined interface and share a common velocity, pressure and turbulence field.

The Unsteady Reynolds Averaged Navier-Stokes (URANS) equations were modelled using the 2^nd^ order Baseline (BSL) Reynolds stress model (RSM) which involved solving an equation for transport of the individual Reynolds stress components. Modelling the exact production term and inherent modelling of stress anisotropies theoretically render the Reynolds stress models more suited to complex flows by naturally including the effects of streamline curvature and sudden changes in strain rate. The flow domain was spatially discretised using solution independent, quasi-structured mesh arrangement comprising of 2.96 × 10^6^ elements. In order to model the boundary layer accurately 1 < *y* + < 6 was enforced across the domain. The boundary condition configuration that was chosen which assigned a mass flow at the inlet and a static pressure condition at the outlet with no-slip boundary condition assigned to the chamber walls. The physical model arrangement analysed possessed an orifice diameter of *d* = 0.067 m, inlet width of *b* = 0.067 m and an inlet radius of *r*_*in*_ = 0.174 m corresponding to an approach flow geometric factor *α* = 2.688.

Three test cases were analysed coinciding with a flow of *Q* = 0.725, 1.677 and 3.111 l/s from which tangential and free-surface profile data was available from the experimental test rig to validate the simulation. The flow field was modelled transiently using an implicit second order accurate time differencing scheme at time steps of 0.01 s. The models were initialised using the results from a steady state model and simulation time was up to 40 seconds. The simulations were computed using a the FIONN supercomputer system at the Irish Centre for High End Computing (ICHEC) with 96 to 120 computer cores taking up to 48 hours to compute. Maximum errors in predicting the primary tangential velocity field and free-surface were found to be in the range of 12% and 22% respectively. Additional information on the simulations performed in this study can be found in^62^.
